# Low B cell counts as risk factor for infectious complications in systemic sclerosis after autologous hematopoietic stem cell transplantation

**DOI:** 10.1186/s13075-020-02255-3

**Published:** 2020-08-08

**Authors:** Michael Gernert, Hans-Peter Tony, Eva Christina Schwaneck, Matthias Fröhlich, Marc Schmalzing

**Affiliations:** Department of Medicine II, Rheumatology and Clinical Immunology, University Hospital of Würzburg, Oberdürrbacher Str. 6, 97080 Würzburg, Germany

**Keywords:** Systemic sclerosis, Autologous hematopoietic stem cell transplantation, Infectious complications, CMV reactivation, B cells

## Abstract

**Background:**

Autologous hematopoietic stem cell transplantation (aHSCT) is a treatment option for a selected group of systemic sclerosis (SSc) patients with good available evidence but can be associated with considerable morbidity and mortality. The aim of this study was to describe infectious complications and distinct immune reconstitution patterns after aHSCT and to detect risk factors in lymphocyte subsets, which are associated with an elevated rate of infections after aHSCT.

**Methods:**

Seventeen patients with SSc were included in this single-center retrospective cohort study. Clinical and laboratory data was collected before and for 12 months after aHSCT, including immunophenotyping of peripheral whole blood by fluorescence-activated cell sorting.

**Results:**

Cytomegalovirus (CMV) reactivations were common in CMV-IgG-positive patients (50%) and needed treatment. Mycotic infections occurred in 17.6%. One patient died (resulting in a mortality of 5.9%) due to pneumonia with consecutive sepsis. All patients showed decreased T helper cells (CD3^+^/CD4^+^) and within the B cell compartment decreased post-switched memory B cells (CD19^+^/CD27^+^/IgD^−^) and elevated naïve B cells (CD19^+^/CD27^−^/IgD^+^) until 12 months after aHSCT. Patients who developed infections had significantly lower B cells before aHSCT than patients who did not develop infections.

**Conclusion:**

After aHSCT, monitoring for infectious complications, especially for CMV reactivations, is crucial as the reconstitution of the immune system takes longer than 12 months. Low peripheral B cells might be a risk factor for an elevated infection rate.

## Background

Systemic sclerosis (SSc) is a fatal autoimmune disease comprising inflammation, vasculopathy, and fibrosis. Due to pulmonary hypertension and lung fibrosis, SSc is the rheumatic disease with the highest case mortality [1]. Evidence for the treatment of SSc is rare, and disease-modifying antirheumatic drugs (DMARDs) are often not effective [2]. Methotrexate can improve skin sclerosis in early diffuse cutaneous forms of SSc [3] and is therefore mentioned in the European League against Rheumatism (EULAR) recommendations [4]. Mycophenolate mofetil [5], cyclophosphamide [6], and rituximab [7, 8] showed some efficacy on disease manifestation. Tocilizumab might have positive effects on lung involvement [9]. The best evidence for effective treatment of SSc is available for autologous hematopoietic stem cell transplantation (aHSCT). Three randomized controlled trials (ASSIST [10], ASTIS [11], and SCOT [12]) have shown superiority of aHSCT versus intravenous cyclophosphamide regarding skin and lung involvement, quality of life, and overall survival. Due to the intensity of the treatment, morbidity and mortality of aHSCT have to be taken into account. A higher rate of infections after aHSCT seems to correlate with CD34^+^ selection [13], which was performed in the three mentioned studies and also in our study.

The aim of the present study was to describe infectious complications appearing after aHSCT of SSc patients and to detect changes in leucocyte subsets during immune reconstitution, which could promote an elevated infection rate after aHSCT.

## Patients and methods

### Patients and monitoring after aHSCT

Seventeen patients who met the ACR/EULAR criteria [14] for SSc and had progressive disease under DMARDs and therefore underwent aHSCT in our center in the years 2009 to 2019 were included. Clinical and laboratory data were taken from the patients’ electronic files (EMIL by itc-ms.de, Marburg, Germany and SAP SE, Walldorf, Germany). Data was collected from 4 months before until 14 months after aHSCT. All infections, which led to a medical consultation in the 14 months after aHSCT, were included. Cytomegalovirus (CMV) DNA and Epstein-Barr virus (EBV) DNA were routinely assessed with PCR for at least 100 days after aHSCT and until T helper cell counts exceeded 200/μl. Reactivations were defined as positive CMV or EBV DNA in the blood in patients, who had a positive CMV- or EBV-IgG before aHSCT.

### Myeloablative autologous hematopoietic stem cell transplantation

Patients were treated analogous to the ASTIS trial protocol [11]: For mobilization of autologous hematopoietic stem cells, patients received 2 g/m^2^ cyclophosphamide with at least 105 μg granulocyte-colony stimulating factor daily, from day 2 after cyclophosphamide, followed by leukapheresis. CD34^+^ selection was performed using immunomagnetic separation (CliniMACS CD34 Complete Kit, Miltenyi Biotec, Bergisch Gladbach, Germany). Due to low stem cell numbers a CD34^+^ selection could not be done in 2 patients. As immunoablative conditioning regimen patients obtained a total of 200 mg/kg body weight (bw) cyclophosphamide on days 1–4 plus a total of 30 mg/kg bw rabbit anti-thymocyte globulin (ATG) on days 2–5. A minimum dose of 2.0 × 10^6^ CD34^+^ autologous hematopoietic stem cells/kg bw was reinfused on day 6.

### Immunophenotyping

Peripheral blood was obtained from all patients before mobilization, which was 8 weeks (median) before application of the immunoablative conditioning regimen and after (at month 1 (range 1–2), month 3 (range 3–4), month 6 (range 6–8), and month 12 (range 10–14)) aHSCT. Immunophenotyping was performed by fluorescence-activated cell sorting using a Navios cytometer (Beckman Coulter, Krefeld, Germany). Three hundred microliters of EDTA-anticoagulated whole blood was immediately processed and incubated with 10 μl of each antibody for 15 min at room temperature. Erythrocytes were lysed with 1.33 ml VersaLyse plus 0.66 ml IOTest3 Fixative Solution (both Beckman Coulter, Krefeld, Germany) for 15 min. Cells were centrifuged for 15 min at 300 RCF; the pellet was resuspended in 2 ml phosphate-buffered saline plus 1% fecal calf serum, centrifuged for 15 min at 300 RCF; and the pellet was resuspended in 300 μl phosphate-buffered saline plus 1% fecal calf serum. The following antibodies were used in different combinations: CD3-PC7, CD4-FITC, CD8-ECD, CD14-PE, CD19-PC7 and CD19-ECD, CD20-APC750, CD27-ECD, CD38-PC5.5, CD45-Krome-Orange and CD45-FITC, CD56/16-PC5 (each Beckman Coulter, Krefeld, Germany), IgD-FITC, CD10-PE (each BD Biosciences, San Jose, CA), CD21-PB (Exbio, Prague, Czech Republic), and IgM-APC (BioLegend, San Diego, CA). By using forward versus sideward scatter, lymphocytes were identified. At least 3000 events within the lymphocyte gate were collected. CD3^+^ events were identified as T cells, CD3^+^/CD4^+^ as T helper cells, CD3^+^/CD8^+^ as cytotoxic T cells, CD56/16^+^ as NK cells, and CD3^+^/CD56/16^+^ as NKT cells. B cells were identified by CD19^+^ positivity. Within the B cell compartment, transitional B cells were defined as CD38^++^/CD10^+^/IgD^+^, pre-switched memory B cells as CD27^+^/IgD^+^, post-switched memory B cells as CD27^+^/IgD^−^, double-negative (DN) B cells as CD27^−^/IgD^−^, and naïve B cells as CD27^−^/IgD^+^.

### Statistical analysis

For testing normal distribution, Shapiro-Wilk tests were performed. Most samples were not normally distributed, so medians with interquartile ranges (IQR) were indicated. To detect differences between paired groups, Wilcoxon signed-rank tests were performed and Mann-Whitney *U* tests for unpaired groups. Excel (Microsoft, Redmond, Washington) was used to collect the data. To perform calculations, SPSS Statistics v 25.0 (IBM, Armonk, NY) was used. Differences were considered significant when two-tailed *P* values were less than 0.05.

## Results

### Patients’ characteristics

Seventeen patients (eight female, nine male; median age 52.0 years, age range 23–64 years) were included in the study. The median disease duration before aHSCT was 3.5 years (range 3 months to 13 years). Six patients were former smokers; one continued smoking through aHSCT. All patients had a diffuse cutaneous form, 15 patients were anti-nuclear antibody positive, eleven patients showed positivity for Scl-70 antibodies, 14 patients had pulmonary fibrosis, and 14 patients had troponin values above the upper limit of normal. Cardiac MRI was done in 13 patients; 6 of them had abnormalities. Twelve patients received a right heart catheterization with 2 of them having a pulmonal arterial hypertension. The median modified Rodnan skin score (mRSS) before aHSCT was 23.0 (range 5–44). The indication for aHSCT was in 41.2% progressive skin involvement, in 35.3% progressive lung involvement and in 23.5% both manifestations. Patients’ characteristics are summarized in Table 1. At baseline, 14 of the 17 patients received an immunosuppressive medication (four patients, prednisolone; one patient, azathioprine; four patients, mycophenolate mofetil; four patients, cyclophosphamide; and one patient, tocilizumab).

**Table 1:** Characteristics of the study population before aHSCT

Characteristics	Values
Female, %	47.1
Age at aHSCT, median (range), years	52.0 (23–64)
Disease duration before aHSCT, median (range), years	3.5 (0.3–13)
Diffuse cutaneous form, %	100
mRSS, median (range) points	23.0 (5–44)
Anti-nuclear antibody positivity, %	88.2
Anti-Scl-70 antibody positivity, %	64.7
Anti-Centromere antibody positivity, %	0
Smoking history, %	
Current	5.9
Ever	41.2
Pulmonary fibrosis on thoracic computed tomography, %	82.4
FVC (% predicted) latest value before aHSCT, median (IQR)	74.0 (58.0–89.0)
DLCO (% predicted) latest value before aHSCT, median (IQR)	44.0 (29.0–59.0)
Troponin positivity, %	82.4
Abnormal 24 h Holter ECG, *n* (%)	1/15 (6.7)
Abnormal cardiac MRI, *n* (%)	6/13 (46.2)
Right heart catheterization, done	12/17
Pulmonal arterial hypertension, *n* (%)	2/12 (16.7)
Mean pulmonal arterial pressure (mPAP), mmHg, median (range)	18.0 (9–30)
Indication for aHSCT	
Skin, %	41.2
Lung, %	35.3
Skin and lung, %	23.5
Positive CMV-Serology, %	35.3
Positive EBV-Serology, %	100

*aHSCT* autologous hematopoietic stem cell transplantation, *CMV* cytomegalovirus, *DLCO* diffusion capacity for carbon monoxide, *EBV* Epstein-Barr virus, *FVC* forced vital capacity, *IQR* inter quartile range, *mRSS* modified Rodnan skin score

### Aciclovir and cotrimoxazole prophylaxes are effective

Patients took aciclovir for 7.5 (IQR 5.8–11.5) months and cotrimoxazole for 9.5 (5.8–14.0) months after aHSCT. Infections with herpes simplex virus or *Pneumocystis jirovecii* did not occur. Prophylaxes were stopped when T helper cells increased over 200/μl or according to the investigators decision, as six patients did not achieve T helper cell counts over 200/μl within the 12 months after aHSCT.

### Infectious complications during the 12 months after aHSCT

Eight patients did not develop any infection in the 12 months after aHSCT (47.1%). Three patients developed mycosis (one CT-morphologic suspected mycotic pneumonia, one esophageal candidiasis, and one oral candidiasis), three patients upper respiratory tract infections, one patient an atypical pneumonia, one patient a pyelonephritis, and one patient a superinfected pancreatic pseudocyst, which required interventional drainage and prolonged antibiotic therapy. One patient died 9 months after aHSCT due to pneumonia with septic shock and lactate acidosis. The mortality rate after aHSCT of SSc patients in our study therefore accounts to 5.9%. All infections that led to a medical consultation are summarized in Table 2. Not included were fevers in aplasia, as it could not be distinguished between an adverse effect of ATG or reconstitution fever or infection. Fever in aplasia occurred in 11 of 17 patients (64.7%).

**Table 2:** Infectious complications, which led to a medical consultation in the 12 months after aHSCT

Infection (causative agent if available)	Frequency	Occurrence after aHSCT, month	Treatment	Treatment duration, weeks
CMV reactivation	3/17 (17.6%); 3/6 of CMV IgG positive (50.0%)	1	Valganciclovir	8 (prophylactic)
		1	Valganciclovir	8 (prophylactic)
		1	Ganciclovir	5
EBV reactivation	1/17 (5.9%)	2	Rituximab 2 × 1 g	2
Mycosis	3/17 (17.6%)			
Nodular pneumonia		1	Voriconazole	5
Oral candidiasis		9	Nystatin	12 (prophylactic)
Esophageal candidiasis		6	Fluconazole	2
Upper respiratory tract infection	3/17 (17.6%)	9	Non	na
		1	Azithromycin	24 (prophylactic)
		10	Amoxicillin/clavulanic acid	1
Atypical pneumonia	1/17 (5.6%)	3	Piperacillin/tazobactam + linezolid	2
Pyelonephritis (*Escherichia coli*)	1/17 (5.9%)	11	Ciprofloxacin	1
Superinfected pancreatic pseudocyst (*Streptococcus anginosus* + *Enterococcus faecalis* and *cloacae* + *Klebsiella pneumoniae*)	1/17 (5.9%)	3	Ceftriaxone + metronidazole, meropenem, ciprofloxacin; drainage, piperacillin/tazobactam, linezolid	14
Lethal pneumonia with lactate acidosis	1/17 (5.9%)	9	Piperacillin/tazobactam	1

*CMV* cytomegalovirus, *EBV* Epstein-Barr virus, *na* not applicable

### CMV and EBV reactivations

A positive CMV serology could be detected in six patients before aHSCT, and three of these patients suffered from a CMV reactivation in the first month after aHSCT. This results in a CMV reactivation rate of 50%. Two of the CMV reactivations were treated orally with valganciclovir, and one patient received intravenous ganciclovir in the intensive care unit. Positive EBV serology was present in all patients before aHSCT, and one patient suffered from an EBV reactivation in month 2, resulting in an EBV reactivation rate of 5.9%. This patient received rituximab, which led to disappearance of EBV DNA in the serum (Table 2).

### Impact of immunosuppression after aHSCT

Administration of DMARDs due to SSc progress after aHSCT did not correlate with higher rates of infections. Six patients received prednisolone (daily dose below 10 mg), six patients received methotrexate, two received hydroxychloroquine, one patient received colchicine plus anakinra, and two patients received rituximab (due to EBV reactivation or pulmonary progress, respectively). Those therapies were given to ten patients, and only two of them (one took prednisolone 5 mg daily, the other methotrexate 15 mg weekly + prednisolone 5 mg daily) developed infections (one esophageal candidiasis and one upper respiratory tract infection, respectively). Immunosuppressive therapy had only a slight impact on the immune reconstitution. Only in month 1 after aHSCT, significant differences could be detected in few leukocyte populations comparing patients taking immunosuppressive therapy (all took prednisolone in a mean dose of 6.25 mg (range 2.5–10 mg); *n* = 8) versus patients without immunosuppressive therapy (*n* = 9): Total T cell percentages were 25.0 (17.3–51.5)% vs 58.0 (50.8–67.8)%; total T cell numbers 161.8 (90.9–514.3)/μl vs 692.1 (443.9–1021.7)/μl; CD8^+^ T cell percentages 24.0 (12.8–34.8)% vs 43.5 (40.5–57.0)/μl; CD8^+^ T cell numbers 127.9 (94.5–341.2)/μl vs 583.7 (332.4–899.9)/μl; NK cell percentages 54.5 (44.5–75.5)% vs 32.5 (21.8–42.8)%.

### Immune reconstitution after aHSCT takes more than 12 months

Leukocytes, neutrophils, and monocytes showed lower numbers per μl in month 12 after aHSCT compared to numbers before aHSCT. The total lymphocyte numbers in month 12 were not different from the baseline total lymphocyte numbers. Regarding the lymphocyte/monocyte ratio, a change from 1.2 (total lymphocytes 1.1 (interquartile range 0.8–1.9) × 10^3^/μl and monocytes 0.9 (0.5–1.1) × 10^3^/μl) before aHSCT towards 2.0 (total lymphocytes 1.2 (0.5–1.5) × 10^3^/μl and monocytes 0.6 (0.5–0.9) × 10^3^/μl) after aHSCT was seen. Within the T cell compartment, aHSCT caused long-lasting total T cell and T helper cell reductions. T cell percentages (within the lymphocyte gate) and absolute numbers showed a long-term decrease until month 12 after aHSCT. Also, the T helper cell percentages and absolute numbers were decreased until month 12 starting from month 1 after aHSCT. Hence, the CD4/CD8 ratio decreased until month 12 (Table 3 and graphically as additional Fig. S1).

**Table 3:** Reconstitution of peripheral blood cells in percentages and absolute cell numbers per μl after aHSCT

	Percentages % (IQR)					Numbers/μl × 10^3^ (IQR)				
	Before aHSCT	Month 1	Month 3	Month 6	Month 12	Before aHSCT	Month 1	Month 3	Month 6	Month 12
Leucocytes	na	na	na	na	na	9.8 (IQR 8.4–13.6)	10.7 (8.3–12.2)	7.6 (5.4–10.5)	7.1 (6.4–8.5)^**†**^	7.1 (5.8–9.1)^**†**^
Neutrophils	77.5 (67.8–83.5)	75.0 (70.5–82.0)	77.0 (69.8–86.3)	79.0 (65.5–82.0)	71.5 (62.0–79.3)	8.5 (6.1–10.8)	7.9 (5.9–9.7)	6.0 (4.3–8.0)	5.6 (4.3–6.4)^**†**^	5.2 (3.7–7.1)^**†**^
Lymphocytes	11.5 (7.3–18.0)	9.5 (5.3–14.8)	10.0 (6.8–17.3)	12.0 (6.5–22.0)	15.5 (11.0–21.0)	1.1 (0.8–1.9)	1.0 (0.6–1.4)	0.7 (0.5–1.0)^**†**^	0.9 (0.5–1.6)^**†**^	1.2 (0.5–1.5)
Monocytes	8.5 (5.3–10.8)	9.0 (6.3–13.0)	10.0 (5.0–13.3)	9.0 (7.5–12.0)	8.5 (7.3–10.0)	0.9 (0.5–1.1)	1.0 (0.6–1.4)^**†**^	0.6 (0.5–0.9)	0.6 (0.4–1.0)^**†**^	0.6 (0.5–0.9)^**†**^
	% within the lymphocyte gate					Numbers/μl				
T cells CD3^+^	72.0 (58.5–78.8)	51.5 (23.0–63.3)^**†**^	37.0 (33.0–55.5)^**†**^	46.0 (32.4–68.0)^**†**^	60.0 (37.5–62.5)^**†**^	745.5 (462.0–1593.7)	473.1 (150.5–837.6)	257.4 (187.5–433.4)^**†**^	325.7 (165.7–796.2)^**†**^	539.4 (237.2–775.6)^**†**^
T helper cells CD3^+^/CD4^+^	50.0 (37.0–57.0)	8.5 (4.3–11.8)^**†**^	7.0 (4.5–10.8)^**†**^	11.0 (5.3–17.3)^**†**^	20.0 (15.0–26.5)^**†**^	605.9 (325.6–829.0)	78.3 (36.7–160.7)^**†**^	49.2 (28.4–95.2)^**†**^	109.4 (26.7–202.1)^**†**^	201.6 (75.8–396.9)^**†**^
Cytotoxic T cells CD3^+^/CD8^+^	14.0 (9.2–21.0)	39.0 (22.0–49.3)^**†**^	28.5 (19.3–34.3)^**†**^	20.5 (15.5–51.8)^**†**^	32.0 (19.5–44.5)^**†**^	146.3 (82.2–286.2)	335.7 (112.8–736.0)	174.8 (115.8–289.4)	140.9 (119.9–543.2)	228.3 (147.1–515.2)
CD4^+^/CD8^+^ ratio	3.4 (1.8–5.2)	0.3 (0.2–0.4)^**†**^	0.3 (0.2–0.4)^**†**^	0.3 (0.2–0.9)^**†**^	0.5 (0.4–1.1)^**†**^	na	na	na	na	na
NK cells CD56/CD16^+^	17.0 (6.0–20.0)	43.5 (25.5–55.8)^**†**^	33.5 (22.8–44.3)^**†**^	24.0 (17.3–36.0)^**†**^	18.0 (10.0–35.5)^**†**^	193.6 (103.4–247.5)	325.4 (240.8–534.9) ^**†**^	205.2 (149.5–346.5)	196.9 (134.3–309.1)	216.1 (123.1–283.0)
NKT cells CD3^+^/CD56/CD16^+^	1.8 (1.0–3.2)	2.0 (0.4–3.2)	2.1 (0.2–3.2)	1.1 (0.4–3.4)	1.6 (1.0–2.2)	28.6 (10.3–50.8)	12.8 (4.6–33.7)	13.6 (3.5–23.6)	8.8 (4.8–32.1)^**†**^	11.7 (5.8–26.6)
Total B cells CD19^+^	5.9 (4.9–15.0)	1.2 (0.3–3.4)^**†**^	13.5 (1.4–29.0)	22.0 (3.8–32.5)^**†**^	24.0 (11.9–31.3)^**†**^	83.2 (47.8–136.7)	7.9 (1.4–50.1)^**†**^	138.9 (10.4–238.1)	215.0 (20.8–291.3)	291.0 (148.9–379.4)^**†**^
	% within the CD19^+^ compartment									
Transitional B cells CD38^++^/CD10^+^/IgD^+^	1.0 (0.3–6.7)	55.0 (7.5–81.8)^**†**^	17.7 (13.4–50.4)^**†**^	5.8 (3.5–19.3)^**†**^	6.2 (1.6–16.1)^**†**^	1.1 (0.2–15.7)	4.2 (0.1–12.5)	28.2 (3.4–47.7)^**†**^	9.7 (2.0–92.1)^**†**^	10.7 (5.5–49.6)^**†**^
Double negative B cells CD27^−^/IgD^−^	3.1 (2.5–6.4)	3.9 (2.0–16.1)	0.8 (0.5–1.8)^**†**^	1.4 (0.6–2.2)^**†**^	1.6 (1.4–2.5)^**†**^	3.6 (1.5–8.4)	1.2 (0.1–1.7)^**†**^	1.2 (0.6–2.3)^**†**^	2.0 (0.9–4.6)	5.8 (3.4–8.8)
Post-switched memory B cells CD27^+^/IgD^−^	9.2 (4.1–18.3)	8.4 (0.9–55.3)	0.9 (0.5–1.9)^**†**^	1.7 (0.5–5.0)^**†**^	2.6 (1.1–2.9)^**†**^	6.6 (4.3–13.1)	0.6 (0.2–1.0)^**†**^	1.3 (0.4–2.2)^**†**^	2.7 (0.6–5.2)^**†**^	5.8 (3.1–10.7)
Pre-switched memory B cells CD27^+^/IgD^+^	6.4 (3.0–10.8)	4.0 (2.3–7.5)	2.5 (1.7–6.3)	2.9 (2.1–4.1)^**†**^	2.1 (1.8–4.2)^**†**^	4.5 (1.8–10.8)	0.2 (0.1–1.7)^**†**^	4.4 (0.9–6.0)^**†**^	4.6 (1.1–11.0)	7.2 (4.4–11.3)
Naïve B cells CD27^−^/IgD^+^	77.0 (60.0–87.2)	76.6 (5.7–92.9)	95.3 (88.4–96.4)	93.8 (89.5–96.2)^**†**^	92.4 (91.2–96.2)^**†**^	58.9 (32.4–130.5)	6.1 (1.2–38.1)^**†**^	127.4 (28.3–238.0)	212.1 (44.1–285.2)^**†**^	293.0 (180.8–353.2)^**†**^

Shown are medians (interquartile range); *na* not applicable; *n* = 10–17

^†^Significant (*P* < 0.05) in a Wilcoxon signed-rank test compared to values before aHSCT

### aHSCT causes a reset of the B cell compartment

The total B cell percentages and numbers were increased in month 12 after aHSCT compared to baseline values. Regarding B cell subsets, in the early phase of the B cell repopulation, transitional B cells were increased. Long-lasting changes were seen with reduced post-switched memory B cell numbers and increased naïve B cell numbers in month 12 after aHSCT compared to values before aHSCT (Table 3 and additional Fig. S1).

### Low B cells before aHSCT are associated with more infections after aHSCT

**Fig. 1:**
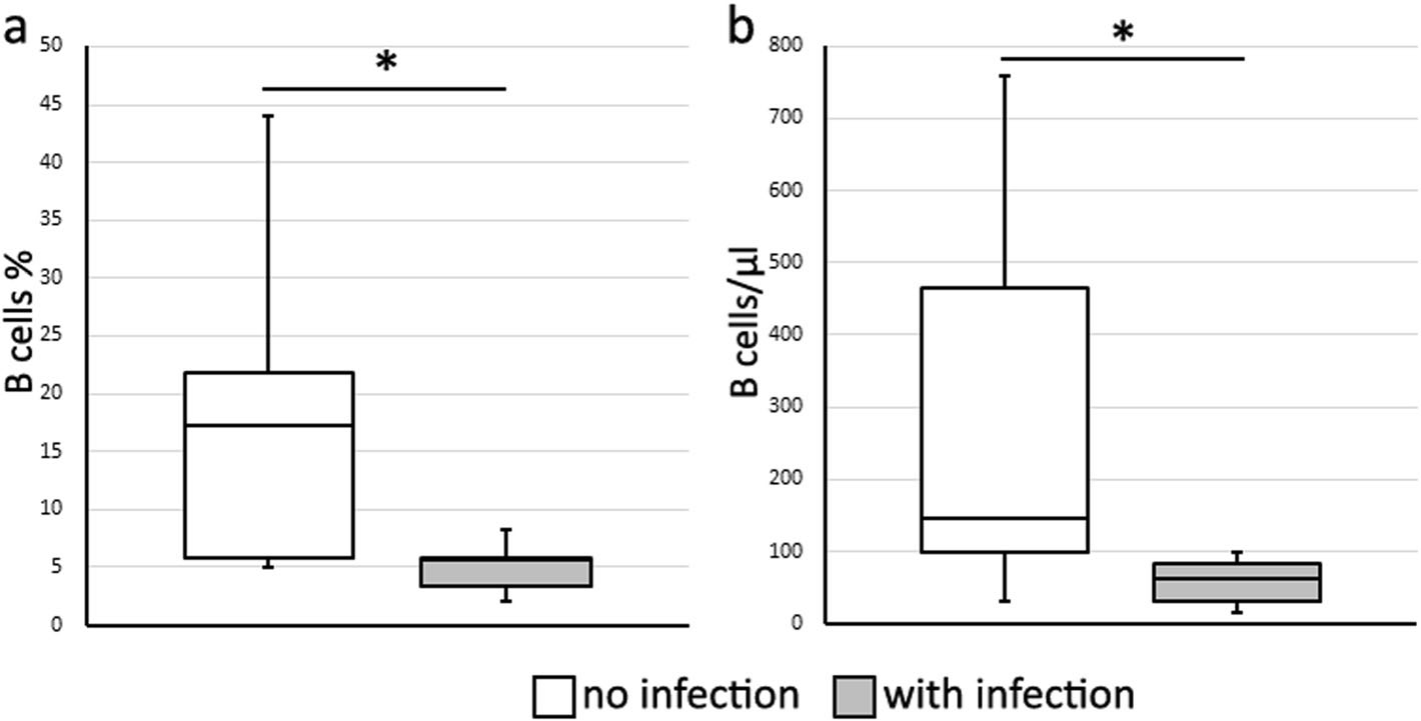
B cell percentages (**a**) and B cell numbers per μl (**b**) of patients who did not develop infections after aHSCT (white boxes) and those who developed at least one infection (gray boxes). Shown are medians with interquartile ranges; whiskers indicate minimums and maximums; *P < 0.05

Comparing patients who developed any kind of infection after aHSCT with those who did not develop infections showed a significant difference in B cell percentages (5.7 (3.3–5.9) % vs. 17.2 (5.9–21.9) %; *P* = 0.012) and B cell numbers (62.2 (30.3–83.2)/μl vs. 146.9 (98.0–465.0)/μl; *P* = 0.012) before aHSCT (Fig. 1). Immunosuppressive medication, in particular cyclophosphamide application or mycophenolate mofetil intake at baseline, did not cause significant differences in B cell percentages or B cell numbers.

Regarding solely virus reactivations (EBV and CMV), no differences within the lymphocyte subsets could be detected comparing patients which suffered from virus reactivations versus those who did not. No significant differences of the B cell numbers and B cell percentages between CMV serology positive and CMV serology negative patients were present.

## Discussion

In this study, we describe a cohort of 17 SSc patients who underwent aHSCT and received a treatment regimen analogous to the ASTIS trial [11]. Thereby, we focused on infections occurring in the 12 months after aHSCT and described the accompanying immune reconstitution.

The immune reconstitution pattern in our cohort showed long-term decreased CD4^+^ T cells, which puts patients at risk for reactivations of herpes viruses and opportunistic infections [15, 16], although opportunistic infections like *Pneumocystis jirovecii* pneumonias are scarce after aHSCT under prophylaxis [17]. In concordance with that, in our study, CMV reactivations were relevant complications. CMV seropositive patients developed CMV viremia in 50% and needed treatment. Determination of the CMV and also of the EBV serostatus before aHSCT therefore seems advisable to identify patients at risk for virus reactivations. This should also comprise DNA measurements after aHSCT. Other groups reported a CMV infection rate between 10 and 64% often without indicating the serostatus for CMV before aHSCT [10, 13]. In the ASTIS trial, the CMV serostatus was determined before aHSCT and the CMV reactivation rate was amounted to 18.7% [11]. The higher CMV reactivation rate in our study might be explained by the usage of a higher total ATG amount for conditioning regimen. A CMV prophylaxis so far is commonly not done. Prophylactic valganciclovir application may be considered for patients at risk temporary after aHSCT as all reactivations occurred within the first month after aHSCT. Valganciclovir may then be switched to aciclovir as long-term intake of valganciclovir can cause cytopenias.

Prophylactic treatment with aciclovir and cotrimoxazole is standard of care after aHSCT and is effective as no patient developed herpes simplex infections or pneumonia due to *Pneumocystis jirovecii* in our study. It is unclear, when these prophylaxes should be stopped. Cotrimoxazole prophylaxis is usually stopped when T helper cells exceed 200 cells/μl [18], but this occurs in only few patients within the first 12 months after aHSCT. Therefore, a stepwise reduction of aciclovir and cotrimoxazole seems feasible. In our cohort, aciclovir was stopped 7.5 months and cotrimoxazole 9.5 months after aHSCT.

Other infections in our cohort were within the spectrum of infections reported in larger SSc cohorts after aHSCT (the main infectious load was due to respiratory tract infections and pneumonias) with an overall infection rate between 58.8 and 75% [11, 12, 19]. In contrast to the other cohorts, we did not detect varicella zoster virus infections or reactivations.

Many patients (64.7%) developed fever during aplasia, but a definite infection mostly could not be proven. Those episodes were not included in our study, especially since fevers due to ATG use or due to leukocyte reconstitution could not be excluded as cause of the fever.

The mortality rate in our study after aHSCT with 5.9% is in the range of the reported studies in the first year after aHSCT. No deaths were reported in the SCOT trial [12], 6.3% in the European society for blood and marrow transplantation (EBMT) register [19], and 10.1% in the ASTIS trial [11]. Differences might be explained by different baseline characteristics among the study populations (in the SCOT trial, no patient had cardiac involvement or pulmonal arterial hypertension [12]) and different myeloablative protocols.

DMARD therapy after aHSCT did not correlate with an increased frequency of severe infections indicating that the aHSCT per se evokes the increased infection rate especially by changing the lymphocyte subsets. Apart from lymphocytes, monocytes play a pathogenetic role in SSc. Increased circulating monocyte numbers compared with healthy controls are reported in SSc patients [20, 21]. We found a reduction of monocyte numbers after aHSCT and a normalization of the lymphocyte/monocyte ratio. Those changes in the lymphocyte subsets and monocytes might promote infectious complications after aHSCT but may be also responsible for the positive effects of aHSCT by resetting the immune system towards normal leukocyte distributions.

The immune reconstitution after aHSCT we present comprises increased B cell numbers, mainly due to increased naïve B cells (accompanied by decreased memory B cells) and decreased CD4^+^ T cells 1 year after aHSCT. This reconstitution pattern is comparable with previously reported SSc cohorts [22, 23], even if different myeloablative protocols (without ATG) were used [23], and is in concordance with the reconstitution described in patients with systemic lupus erythematodes [24] and multiple sclerosis [25]. In contrast, patients with rheumatoid arthritis showed normalized levels of B cells 1 year after aHSCT, although a similar myeloablative protocol was performed [26]. It could be speculated that the different reconstitution patterns might be influenced by different underlying autoimmune diseases. However, the long-term reduction of CD4^+^ T cells seems to be a common finding after aHSCT as it is also reported in malignant diseases [27, 28].

Low B cells before aHSCT were associated with more infections after aHSCT which might lead to a careful monitoring of patients who initially have low B cells. The predictive value of B cells has been investigated in septic patients. In a meta-analysis, sepsis non-survivors had reduced B cell numbers at the onset of sepsis compared to sepsis survivors [29].

To our knowledge, this is the first description of the potential predictive significance of B cells for outcomes after autologous hematopoietic stem cell transplantation. Confounders for baseline B cell percentages and B cell numbers, like different underlying autoimmune diseases or the influence of the pre-transplant treatment, should be evaluated in bigger cohorts in the future.

Our study is limited because of its retrospective design and the low number of patients. Two of our patients did not receive CD34^+^ selection of their autologous hematopoietic stem cells, which might have influenced the course of their immune reconstitution.

## Conclusion

Our data suggest that it is advisable to test all SSc patients before and after aHSCT for CMV. Especially patients who have low B cells before aHSCT might be at risk for the development of infections. Our data of median lymphocyte percentages and numbers can be used as reference values after aHSCT to assess the reconstitution state in a transplanted SSc patient.

## Supplementary information

**Additional file 1: Fig. S1.** Reconstitution of peripheral blood cells. Boxplots show values at baseline, month 1, month 3, month 6 and month 12 of (a) neutrophil, lymphocyte and monocyte percentages, (b) leukocyte, neutrophil, lymphocyte and monocyte numbers/μl, (c) total B cell and naïve B cell percentages, (d) total B cell and naïve B cell numbers /μl, (e) transitional, double negative, post-switched memory and pre-switched memory B cell percentages, (f) transitional, double negative, post-switched memory and pre-switched memory B cell numbers /μl, (g) total T cell, CD4^+^ T cell, CD8^+^ T cell, NK cell and NKT cell percentages and (h) total T cell, CD4^+^ T cell, CD8^+^ T cell, NK cell and NKT cell numbers/μl. Boxplots show medians with 25th and 75th percentiles, whiskers indicate minimums and maximums, respectively. White blots indicate cell percentages, gray blots indicate cell numbers/μl. * significant difference compared to baseline value, *P* < 0.05.

## Data Availability

The data used and/or analyzed during the current study are available from the corresponding author on reasonable request.

## Abbreviations

ACR: American College of Rheumatology
aHSCT: Autologous hematopoietic stem cell transplantation
APC: Allophycocyanin
ASSIST: American Scleroderma Stem Cell versus Immune Suppression Trial
ASTIS: Autologous Stem cell Transplantation International Scleroderma trial
ATG: Anti-thymocyte globulin
bw: Body weight
CD: Cluster of differentiation
CMV: Cytomegalovirus
CT: Computed tomography
dcSSc: Diffuse cutaneous form of systemic sclerosis
DMARDs: Disease-modifying anti rheumatic drugs
ECD: Phycoerythrin-Texas Red
EBMT: European Society for Blood and Marrow Transplantation
EBV: Epstein-Barr virus
EDTA: Ethylenediaminetetraacetic acid
EULAR: The European League Against Rheumatism
FITC: IgD-fluorescein isothiocyanate
Ig: Immunoglobulin
IQR: Interquartile range
mRSS: Modified Rodnan skin score
na: Not applicable
PB: Pacific Blue
PC: Phycoerythrin-cyanin
PE: Phycoerythrin
RCF: Relative centrifugal field
SCOT: Scleroderma: Cyclophosphamide or Transplantation trial
SSc: Systemic sclerosis
