# Supplementary figures and images for: Low B cell counts as risk factor for infectious complications in systemic sclerosis after autologous hematopoietic stem cell transplantation
Source: Arthritis Res Ther. 2020 Aug 8;22:183. doi: 10.1186/s13075-020-02255-3 (PMC7414656; doi:10.1186/s13075-020-02255-3)

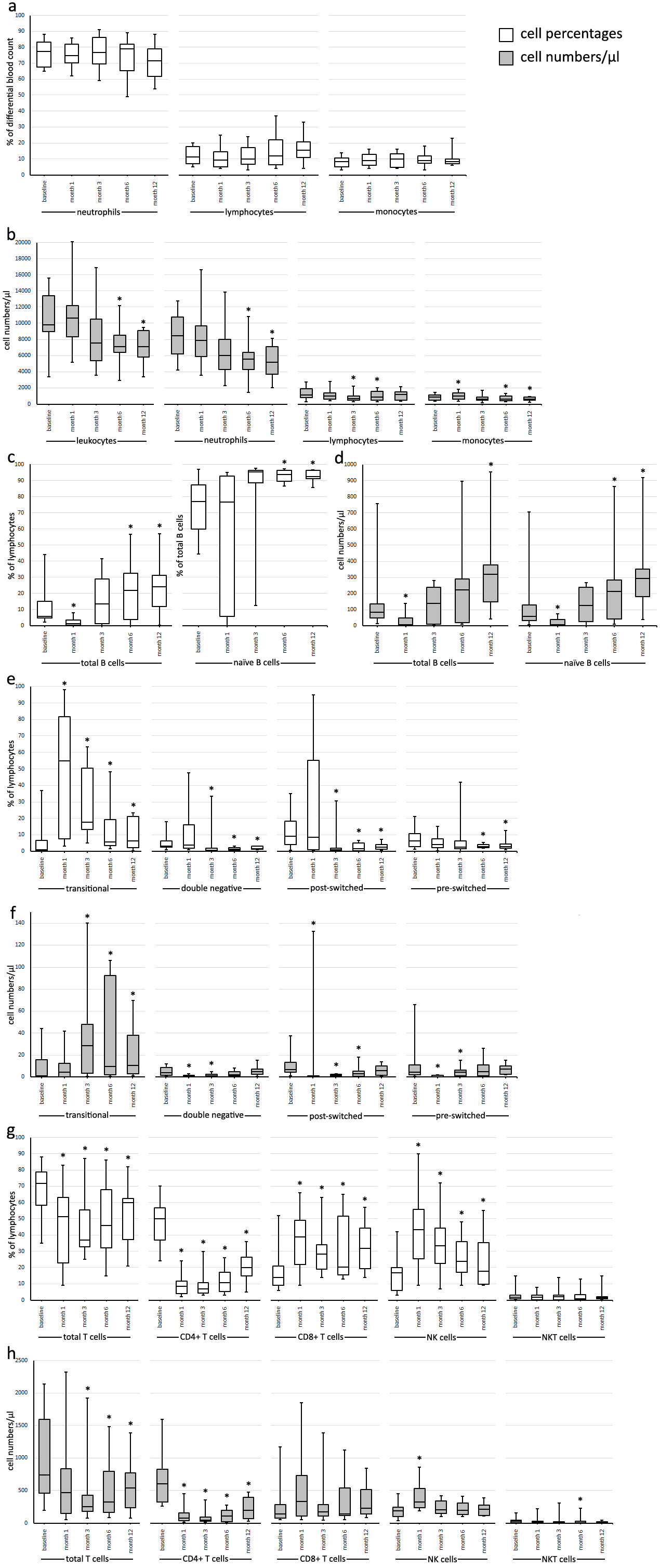

Supplement: Supplementary file 1 — Additional file 1: Fig. S1. Reconstitution of peripheral blood cells. Boxplots show values at baseline, month 1, month 3, month 6 and month 12 of (a) neutrophil, lymphocyte and monocyte percentages, (b) leukocyte, neutrophil, lymphocyte and monocyte numbers/μl, (c) total B cell and naïve B cell percentages, (d) total B cell and naïve B cell numbers /μl, (e) transitional, double negative, post-switched memory and pre-switched memory B cell percentages, (f) transitional, double negative, post-switched memory and pre-switched memory B cell numbers /μl, (g) total T cell, CD4+ T cell, CD8+ T cell, NK cell and NKT cell percentages and (h) total T cell, CD4+ T cell, CD8+ T cell, NK cell and NKT cell numbers/μl. Boxplots show medians with 25th and 75th percentiles, whiskers indicate minimums and maximums, respectively. White blots indicate cell percentages, gray blots indicate cell numbers/μl. * significant difference compared to baseline value, P < 0.05. [file 13075_2020_2255_MOESM1_ESM.tif]
